# Laser-Assisted Surgical Management of Ankyloglossia in Mixed Age Groups: A Case Series With Eight-Month Follow-Up

**DOI:** 10.7759/cureus.107727

**Published:** 2026-04-26

**Authors:** Anand Suresh, Sanjeev Ravindran, Shyamala Devi

**Affiliations:** 1 Periodontics, PSM College of Dental Science and Research, Thrissur, IND

**Keywords:** ankyloglossia, diode laser, frenectomy, kotlow classification, minimally invasive periodontal therapy

## Abstract

Ankyloglossia restricts tongue mobility and may impair speech, oral hygiene, and quality of life. Lasers offer advantages over scalpel techniques, including hemostasis and improved patient comfort. This report presents three cases of Kotlow Class II and III ankyloglossia treated with diode laser frenectomy across different age groups, with an eight-month follow-up. A 12-year-old boy (Class III), a 20-year-old female (Class III), and a 24-year-old male (Class II) underwent diode laser frenectomy. Postoperative care included analgesics and tongue exercises, and functional assessment was carried out up to eight months. All cases demonstrated improved tongue mobility, protrusion, and speech clarity, with minimal postoperative pain and no complications. The outcomes remained stable throughout the follow-up period. Diode laser frenectomy appears to be a predictable and minimally invasive treatment option for ankyloglossia in adolescents and adults, providing favorable long-term results.

## Introduction

The lingual frenum is a mucosal fold that connects the ventral surface of the tongue to the floor of the mouth and plays a crucial role in stabilizing tongue movements required for speech, mastication, and swallowing [1]. Ankyloglossia, commonly referred to as tongue-tie, is a congenital oral anomaly characterized by a short, thick, or fibrotic lingual frenulum that restricts tongue mobility and may interfere with normal oral function [2]. The reported prevalence ranges from 4% to 11%, with variability attributed to differences in diagnostic criteria, age groups studied, and methods of clinical assessment [3].

Clinically, ankyloglossia presents with a spectrum of functional limitations depending on severity and age. In infants, it is frequently associated with breastfeeding difficulties, whereas, in children and adults, it may result in speech articulation problems, compromised oral hygiene, periodontal issues, and psychosocial discomfort [4,5]. Accurate diagnosis and grading of severity are essential for appropriate treatment planning. Among the available systems, Kotlow's classification is widely used due to its simplicity and its ability to correlate anatomical restriction with functional impairment [5]. However, despite its clinical utility, variability in assessment criteria and lack of universally accepted functional scoring systems remain challenges in standardizing diagnosis and outcome evaluation.

Surgical intervention is generally indicated in moderate-to-severe cases associated with functional impairment. Conventional scalpel frenectomy has long been considered the standard treatment modality; however, it is often associated with intraoperative bleeding, need for suturing, postoperative discomfort, and delayed healing [6]. These limitations have prompted the exploration of minimally invasive alternatives, particularly laser-assisted techniques.

Various laser systems, including diode, carbon dioxide (CO₂), and erbium-based lasers, have been utilized in oral soft tissue procedures. Each modality offers distinct advantages based on wavelength characteristics and tissue interaction. CO₂ lasers provide excellent superficial ablation with strong hemostatic properties, while erbium lasers allow precise cutting with minimal thermal damage but comparatively less coagulation [7,8]. Diode lasers, owing to their high affinity for hemoglobin and melanin, offer effective coagulation, reduced intraoperative bleeding, and improved surgical visibility, making them particularly suitable for soft tissue procedures, such as frenectomy [7-9].

Laser-assisted frenectomy has been increasingly advocated as a minimally invasive alternative due to its advantages, including reduced operative time, minimal postoperative pain, absence of sutures, and faster healing [7,9,10]. However, despite these benefits, certain limitations must be acknowledged. These include higher equipment costs, technique sensitivity, and variability in clinical outcomes depending on operator experience and laser parameters [6,10]. Furthermore, existing literature is predominantly composed of case reports and small case series, with limited long-term follow-up data and a lack of standardized outcome measures, particularly across different age groups.

Given these considerations, there remains a need for clinically relevant data evaluating the effectiveness and stability of laser-assisted frenectomy in diverse patient populations. The present case series aims to report the clinical outcomes of diode laser-assisted management of ankyloglossia in mixed age groups, with an eight-month follow-up, focusing on functional improvement, healing characteristics, and procedural predictability.

## Case presentation

Methodology

This case series included three patients diagnosed with ankyloglossia and treated using diode laser-assisted surgery in the Department of Periodontology. Written informed consent was obtained from all patients or their guardians prior to treatment. Preoperative assessment was performed using Kotlow's classification (1999), as elucidated in Table 1.

**Table 1:** Kotlow's classification of ankyloglossia (1999) The free tongue length is measured from the insertion of the lingual frenum to the tip of the tongue. Adapted from Kotlow [5]. Table credits: Dr. Anand Suresh

Class	Severity	Free tongue length
Class I	Mild	12-16 mm
Class II	Moderate	8-11 mm
Class III	Severe	3-7 mm
Class IV	Complete	<3 mm

All procedures were carried out under local anesthesia using a diode laser following standard laser safety protocols. Patients were followed up for a period of eight months to evaluate healing and functional outcomes.

Objective Functional Assessment

In addition to clinical examination and Kotlow's classification, functional assessment was performed based on tongue mobility parameters, including tongue protrusion beyond the vermilion border, elevation toward the palate, and lateral movements. Speech-related limitations were evaluated clinically based on articulation clarity during phonetic pronunciation (particularly lingual consonants, such as "t," "d," "l," and "r"). Due to the clinical setting and case series design, standardized quantitative scoring systems were not employed; however, all patients were assessed consistently using the same clinical criteria at baseline and during follow-up visits to ensure comparability of outcomes.

Case 1

**Figure 1:**
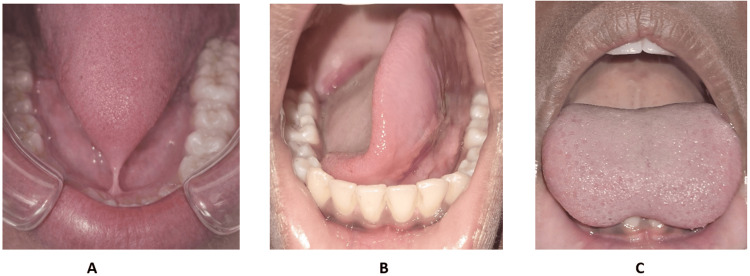
Case 1 (A-C): Preoperative clinical photograph showing restricted tongue protrusion due to ankyloglossia in a 12-year-old patient (Kotlow Class III) Picture credits: Dr. Anand Suresh

A 12-year-old boy presented with difficulty in speech articulation and limited tongue protrusion. Medical and family histories were non-contributory. Clinical examination revealed restricted tongue elevation with blanching of the floor of the mouth during attempted protrusion (as shown in Figure 1).

**Figure 2:**
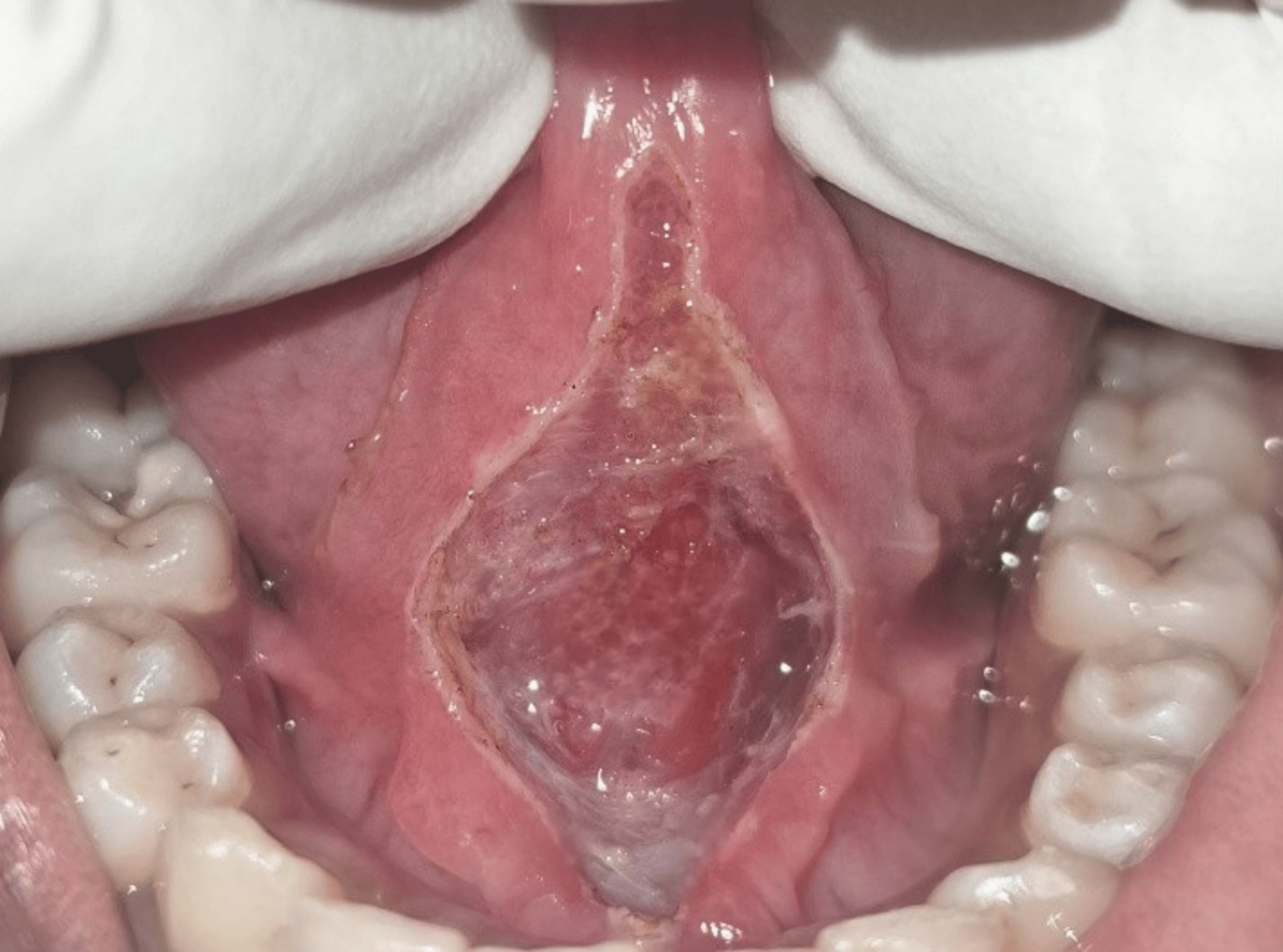
Case 1: Immediate postoperative view following lingual frenectomy performed with a diode laser

Measurement of the free tongue length was 6 mm, indicating Kotlow Class III (severe) ankyloglossia. After obtaining informed consent from the guardian, the procedure was performed under local anesthesia (2% lignocaine with 1:80,000 epinephrine). Standard laser safety protocols were followed. A diode laser (810 nm wavelength, initiated fiber tip, continuous mode at 2 W) was used in contact mode. The fiber tip was positioned at the apex of the frenulum and advanced toward the base using a controlled brushing motion, while gentle traction was applied to the tongue. Adequate release was confirmed by improved tongue elevation and protrusion. This is shown in Figure 2.

**Figure 3:**
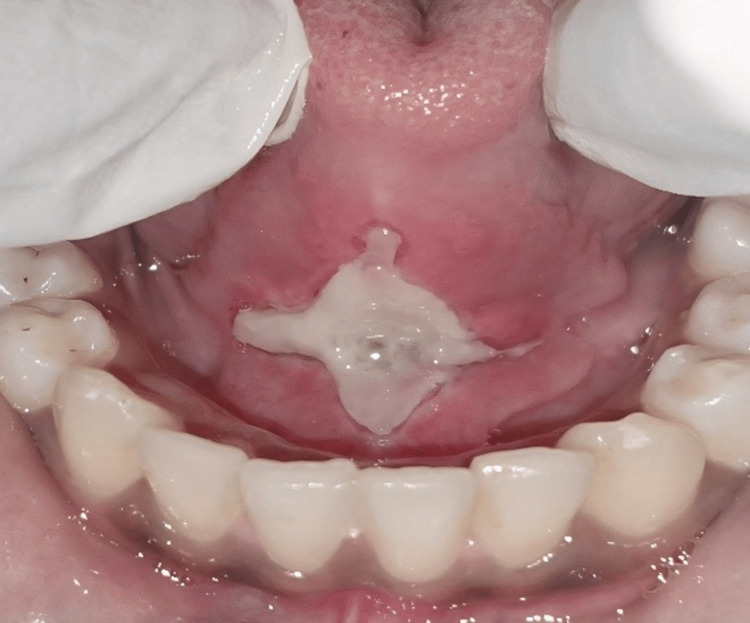
Case 1: Healing observed at one‑week follow‑up

The surgical site exhibited excellent hemostasis, and sutures were not required. Postoperative tongue mobility exercises were prescribed and reviewed after one week, which exhibited an ongoing healing process. This is shown in Figure 3.

**Figure 4:**
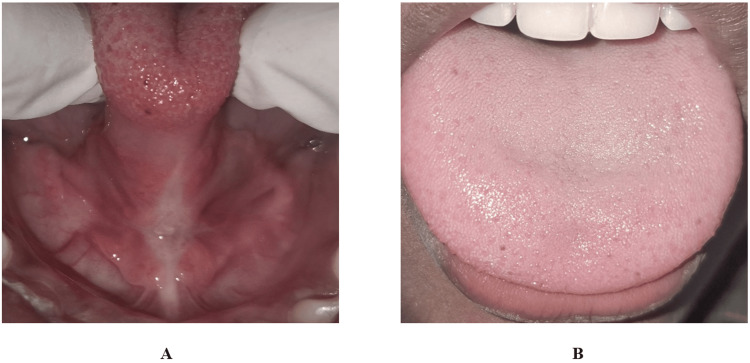
Case 1: (A) Satisfactory and stable wound healing after eight months of follow-up. (B) Follow-up examination after eight months with improved tongue protrusion

At the eight-month follow-up, the patient demonstrated sustained improvement in speech and tongue movement without scarring or recurrence. This is seen in Figure 4.

Case 2

**Figure 5:**
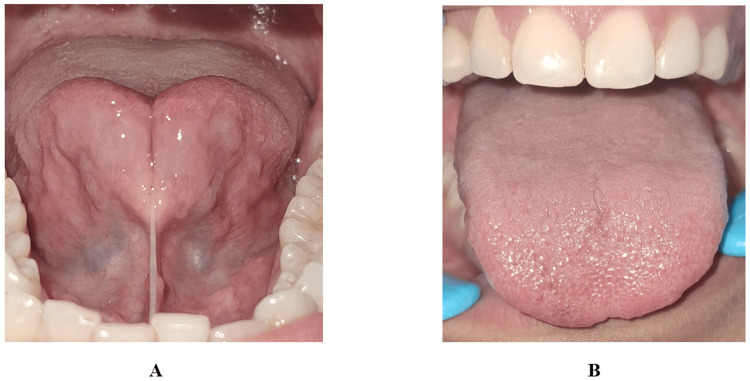
Case 2: (A-B) Preoperative clinical photograph showing restricted tongue protrusion due to ankyloglossia in a 20-year-old female patient (Kotlow Class III)

A 20-year-old female patient reported difficulty in speech pronunciation and discomfort during oral hygiene practices. Intraoral examination revealed a short and fibrotic lingual frenulum with a free tongue length of 9 mm, consistent with Kotlow Class III (severe) ankyloglossia. This is shown in Figure 5.

**Figure 6:**
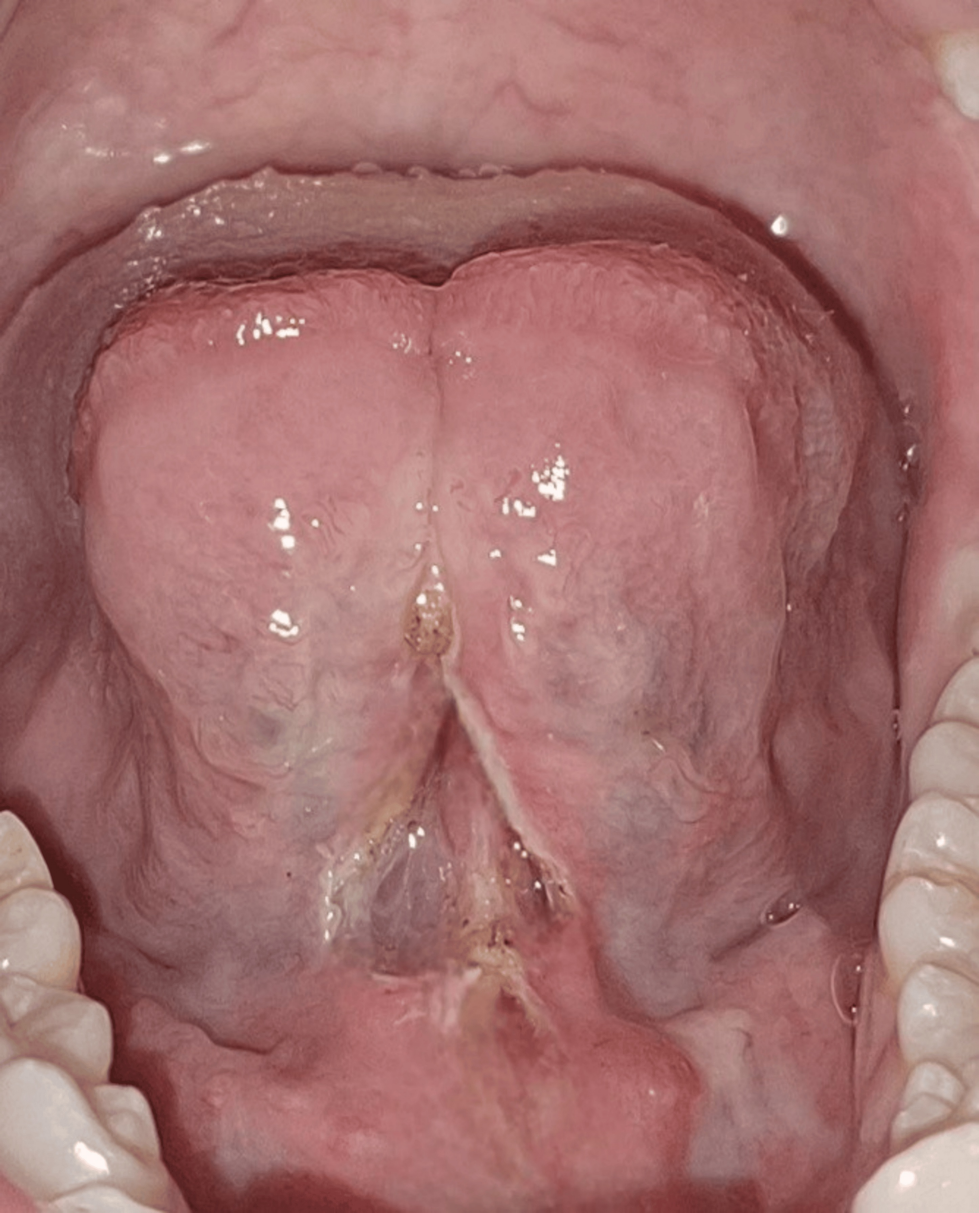
Case 2: Immediate postoperative view of lingual frenectomy with a bloodless area performed with a diode laser

Laser-assisted frenectomy was planned after informed consent. Local infiltration anesthesia was administered, and a diode laser (810 nm, continuous mode at 2 W) was used. A horizontal-to-vertical release was performed to ensure the complete elimination of fibrotic bands while preserving surrounding structures. The procedure was completed with minimal discomfort and negligible bleeding. No sutures were required. This is seen in Figure 6.

**Figure 7:**
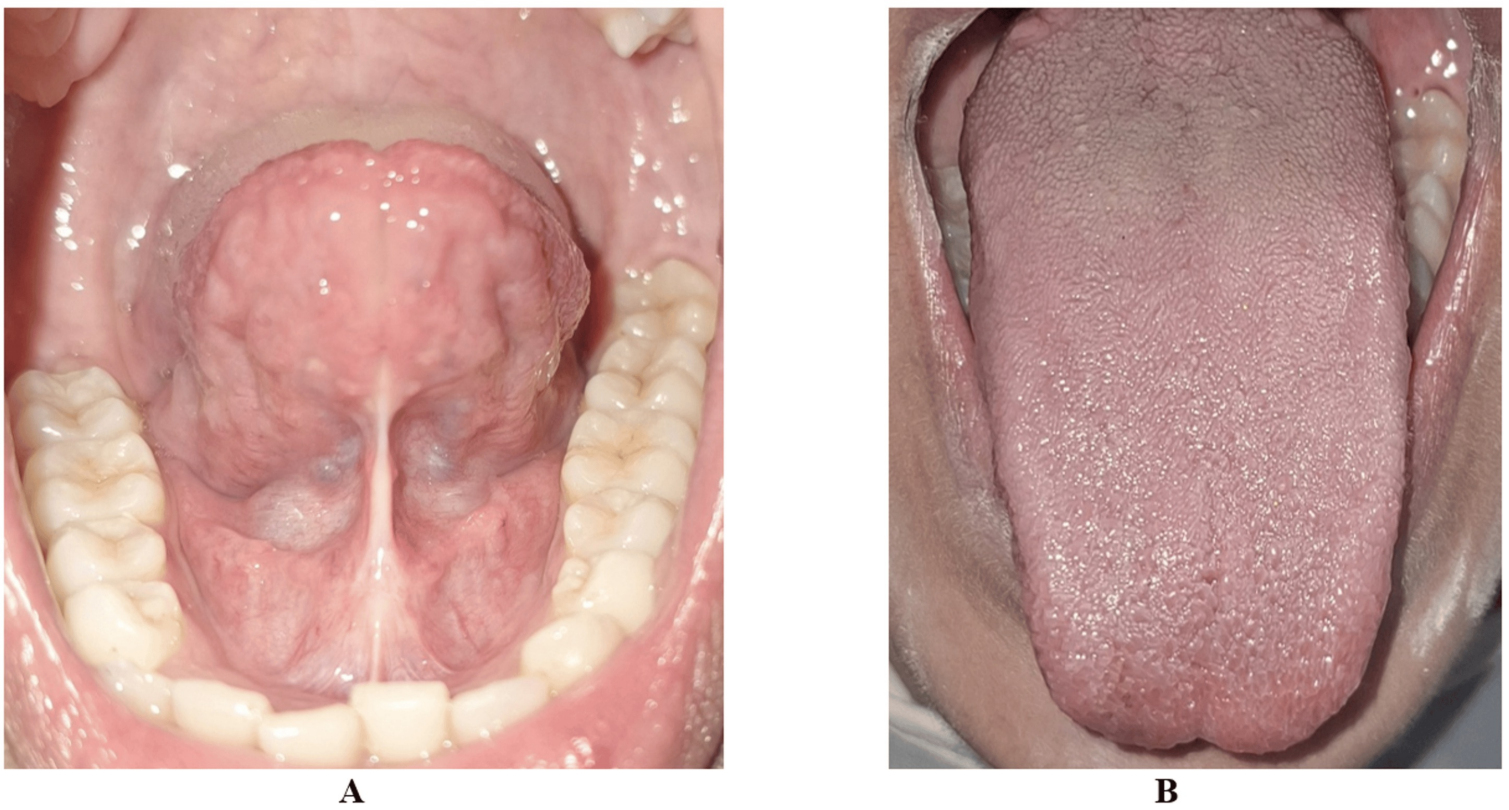
Case 2: (A-B) Postoperative view after eight months with satisfactory and stable wound healing and improved tongue protrusion

Healing was uneventful, and the patient reported minimal postoperative pain. At the eight-month follow-up, marked improvement in tongue mobility, speech articulation, and oral hygiene maintenance was observed, as shown in Figure 7.

Case 3

**Figure 8:**
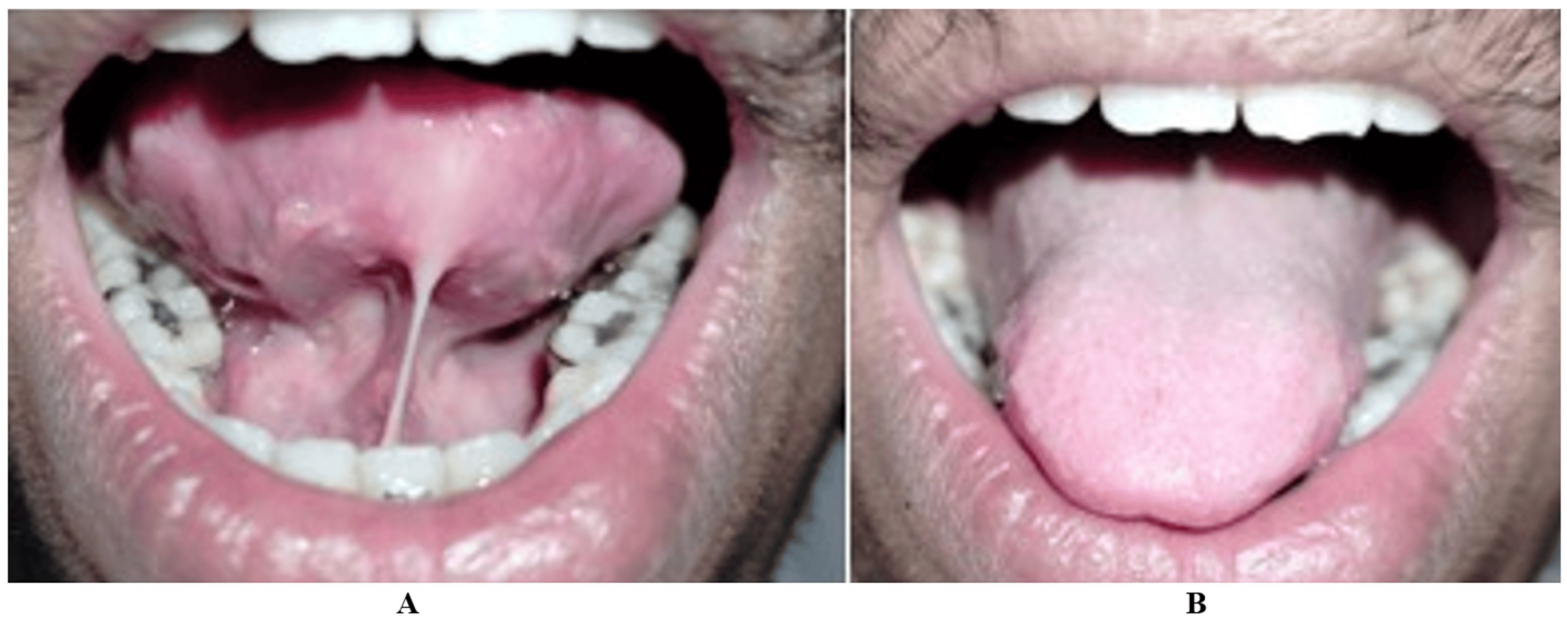
Case 3: (A-B) Preoperative clinical photograph showing restricted tongue protrusion due to ankyloglossia in a 24-year-old male patient (Kotlow Class II)

A 24-year-old male patient presented with restricted tongue elevation and difficulty during speech. Clinical examination revealed a short lingual frenulum with a free tongue length of 10 mm, corresponding to Kotlow Class II (moderate) ankyloglossia (Figure 8).

**Figure 9:**
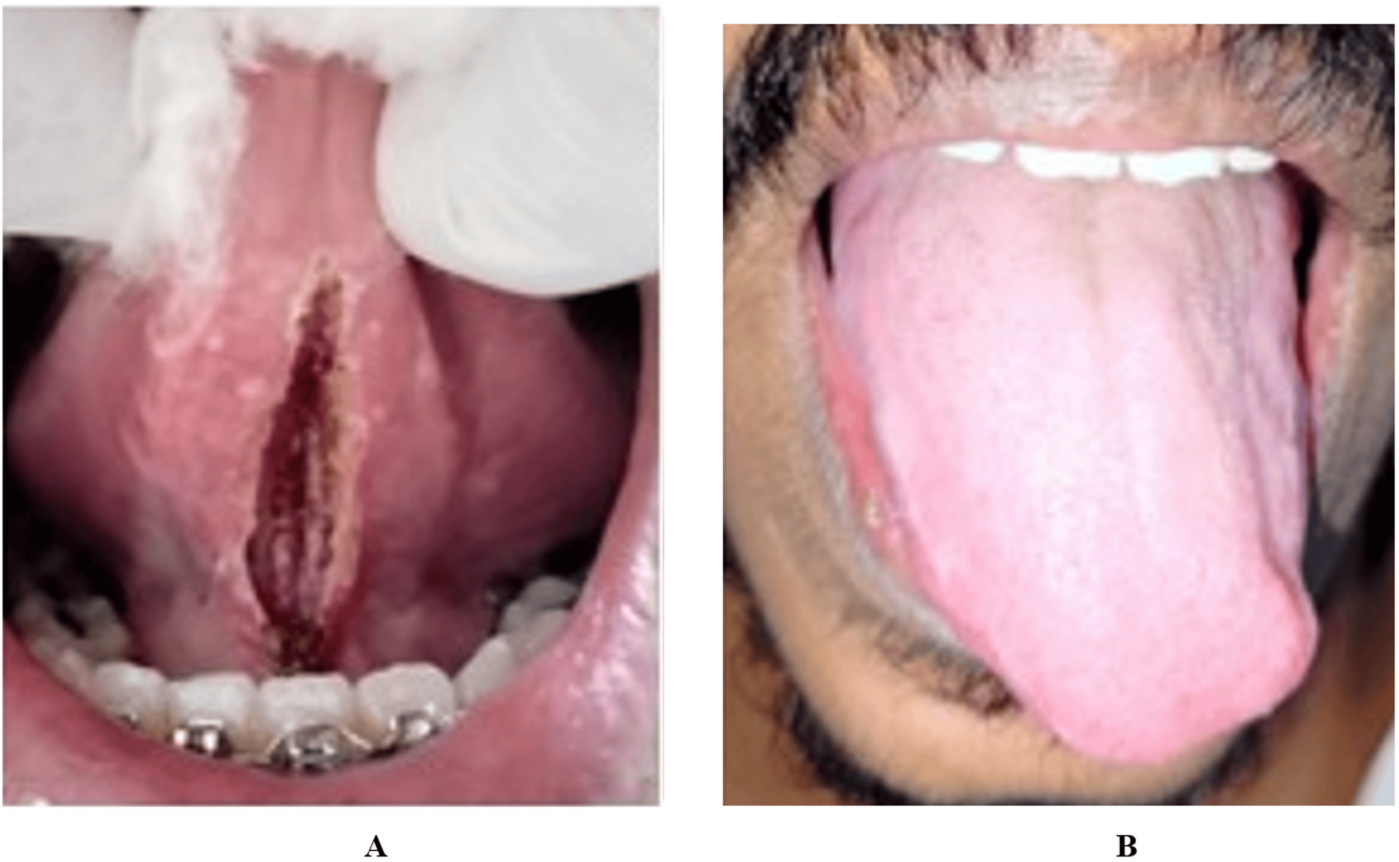
Case 3: (A-B) Post-operative image of lingual frenectomy performed with a diode laser

Under local anesthesia, diode laser frenectomy was performed using an 810 nm laser with an initiated fiber tip in continuous mode at 2 W. The frenulum was released gradually from superficial to deeper fibers using sweeping laser strokes until adequate tongue mobility was achieved. Immediate improvement in tongue elevation was evident intraoperatively. This is seen in Figure 9.

**Figure 10:**
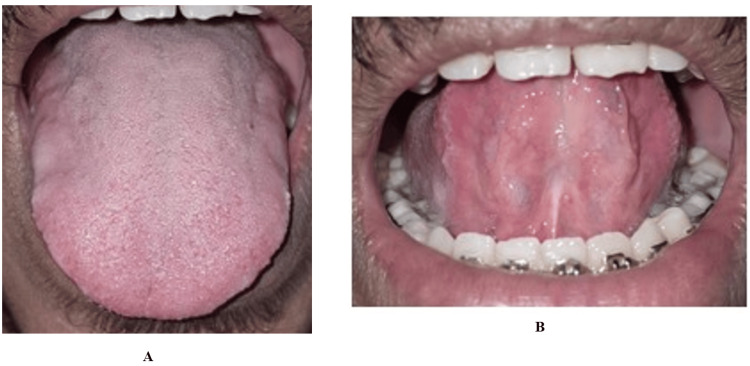
Case 3: (A and B) Postoperative view after eight months with satisfactory and stable wound healing and improved tongue protrusion

The surgical field remained bloodless, and no sutures were placed. At the eight-month review, the patient exhibited stable functional improvement without complications or relapse. This is observed in Figure 10.

## Discussion

Ankyloglossia is a developmental anomaly with a variable clinical presentation depending on anatomical severity and patient age. Functional impairment tends to increase with decreasing free tongue length, particularly in cases classified as Kotlow Classes III and IV, which are often associated with speech difficulties, compromised oral hygiene, and mechanical limitations during mastication and swallowing [4,5].

In the present case series, patients represented mixed age groups with moderate (Class II) and severe (Class III) ankyloglossia. The observed functional limitations - primarily speech impairment and restricted tongue mobility - are consistent with previous clinical reports [8,9]. Notably, the severity of restriction correlated well with the degree of functional compromise, supporting the clinical utility of Kotlow's classification as a diagnostic and treatment-planning tool [5]. This is elucidated in Table 2.

**Table 2:** Summary of case series according to Kotlow's classification Table credits: Dr. Anand Suresh

Case	Age/Sex	Kotlow’s Class	Primary Functional Limitation	Laser Procedure	Outcome at 8 Months
Case 1	12-yr boy	Class III	Speech difficulty, limited protrusion	Diode laser frenectomy	Improved mobility and speech
Case 2	20-yr female	Class III	Speech difficulty, hygiene limitation	Diode laser frenectomy	Marked improvement, no relapse
Case 3	24-yr male	Class II	Restricted elevation, speech discomfort	Diode laser frenectomy	Stable functional outcome

Kotlow's classification was utilized in this study as a practical clinical tool to assess severity and guide treatment planning. The observed correlation between classification grade and functional limitation in our cases supports its continued clinical utility. However, it is important to recognize that Kotlow's classification is primarily anatomical and does not incorporate functional parameters, which may limit its comprehensiveness in outcome assessment. This highlights the need for adjunctive functional evaluation tools in both clinical practice and research settings [4,5].

Laser-assisted frenectomy using a diode laser (810 nm) demonstrated predictable and favorable outcomes across all cases. The advantages observed in this series - minimal intraoperative bleeding, absence of suturing, reduced postoperative discomfort, and rapid healing - are well supported in the literature. Diode lasers exhibit high affinity for hemoglobin and melanin, enabling excellent coagulation and hemostasis, which significantly enhances surgical visibility and precision [7,9,10]. This is particularly beneficial in pediatric and anxious patients, where shorter operative times and reduced invasiveness improve patient compliance.

The postoperative outcomes in this study align with findings by Mezzapesa et al. and Tancredi et al., who reported improved tongue mobility and speech outcomes following diode laser frenectomy, with minimal complications [7,11]. Furthermore, the absence of scar formation and relapse at the eight-month follow-up in the present cases supports the long-term stability of laser-assisted procedures. Early initiation of tongue mobility exercises likely contributed to functional rehabilitation and prevention of reattachment, as emphasized in previous studies [6,12].

Compared to conventional scalpel techniques, which may require sutures and are associated with greater postoperative morbidity, laser-assisted approaches provide a minimally invasive alternative with enhanced patient comfort and faster recovery [6,10]. The bloodless surgical field and reduced need for local anesthesia further contribute to improved clinical efficiency.

Differential Diagnosis

Although the diagnosis of ankyloglossia in the present cases was clinically evident based on restricted tongue mobility and reduced free tongue length, it is important to consider differential diagnoses that may present with similar functional limitations. These include neuromuscular disorders affecting tongue movement, speech articulation disorders unrelated to anatomical restriction, and variations in lingual frenulum attachment without functional impairment. Careful clinical examination and correlation with functional findings are therefore essential to establish an accurate diagnosis and appropriate treatment plan [10-12].

Limitations

The present case series has several limitations that should be considered when interpreting the findings. The small sample size limits the generalizability of the results and restricts the ability to draw definitive conclusions. Additionally, the absence of a control group treated with conventional techniques precludes direct comparison of clinical outcomes between treatment modalities. The study primarily relies on qualitative clinical assessment, and standardized objective evaluation tools - such as validated speech assessment scales or quantitative tongue mobility indices - were not utilized. This may introduce subjectivity in outcome evaluation and limit the precision of functional improvement measurements. Furthermore, although patients were followed for eight months, longer-term follow-up would be beneficial to assess the stability of outcomes and the potential for late recurrence. Operator-dependent factors and variability in laser parameters may also influence clinical outcomes, which should be taken into account when interpreting the reproducibility of the procedure. Future studies incorporating larger sample sizes, randomized controlled designs, and objective outcome measures are warranted to further validate the clinical effectiveness and long-term benefits of diode laser-assisted frenectomy [10,11].

Clinical Implications

Patients with Class II ankyloglossia primarily exhibited speech-related complaints, whereas the Class III case demonstrated more pronounced functional compromise, consistent with previous reports [8,9]. Laser-assisted management proved effective across both moderate-and-severe classes, providing precise tissue release, minimal trauma, and rapid recovery [10]. The absence of sutures and reduced postoperative morbidity improved patient compliance across all age groups. These findings align with existing literature supporting diode laser use as a reliable alternative to conventional scalpel techniques [9,11,12]. Laser-assisted surgical management of ankyloglossia offers a minimally invasive, bloodless, and patient-friendly alternative to conventional techniques, providing predictable functional outcomes across different age groups.

Future Perspectives

Future studies with larger sample sizes, randomized controlled designs, and objective functional outcome measures are recommended to further validate the clinical advantages of diode laser-assisted frenectomy.

## Conclusions

Diode laser-assisted surgical management of ankyloglossia is a safe, minimally invasive, and effective treatment modality applicable across mixed age groups. This case series demonstrates predictable healing, significant functional improvement, and stable outcomes at eight-month follow-up. Laser-assisted frenectomy should be considered a preferred alternative to conventional surgical approaches, particularly in patients seeking minimal discomfort and rapid recovery.
